# On the Employment of Urate of Ammonia, &c.

**Published:** 1854-02

**Authors:** 

**Affiliations:** Tabinzen


Translated from the Gazette des Hopitaux.

*On the employment of Urate of Ammonia in Intermittent fevers and European Cholera Morbus.* By Dr. BAUR, of Tabinzen.

TO THE EDITOR OF THE GAZETTE DES HOPITAUX.

*My Dear Confrere:* I have received from Dr. Baur, of Tabinzen, Wurtemberg, a letter, which, on account of its real interest, I shall be pleased to see inserted in your following number. I have translated it literally from the German.

Accept etc., etc.

SICHEL, D. M. P.

"During the last eight years I have been making observations on the medical properties of *Urate of Ammonia;* these experiments have resulted in the discovery of two positive facts, which are not without their practical value.

In the first place I have found, that the urate of ammonia is an extremely active remedy in intermittent affections, such as intermittent fevers and periodical neuroses. After having assured myself of its effects in many cases, I have during the last summer been induced, by the advice of a physician of the Bavarian Army, to make still further experiments with this remedy in intermittent fever. In a majority of cases it had a happy effect, although at the time of these trials the intensity of the fevers was very much augmented. Ordinarily the urate was administered every two hours in doses of from 10 to 15 centigrammes in the form of a powder with sugar. A second valuable property of this substance is that which I have often proved in European Cholera. In this affection I have employed it generally in the form of lavements in quantity of 25 centigrammes, with a little starch and a half pint of water. It is necessary that the patient continue it sometime and repeat according to circumstances. In European Cholera I have not as yet found it necessary to repeat frequently, one or two applications having always thus far cut short the disease. Instead of the lavement we may use an ointment composed of from two to four grammes of urate of ammonia to thirty grammes of simple cerate. A spoonful of coffee may be rubbed each hour upon the integuments covering the abdomen. It is truly remarkable how rapidly this medicine acts when applied as a wash. It procures immediate ease from the patient, arrests the choleric evacuations, restores the stools to their normal character, renders the skin moist, establishes the pulse, and causes a cessation of the cramps. A similar effect I have seen from no other medicine.

Since, most physicians are more and more convinced that Asiatic cholera differs in no essential or specific character from European cholera, being distinguished mainly by its epidemic development, and its greater intensity, I have for a long time been desirous of experimenting with the urate of ammonia in the more severe form of the disease, but since becoming acquainted with the virtues of this remedy, I have not seen a single case of epidemic cholera. Relying on your friendship, I must therefore request you to make some experiment yourself, or to make an appeal to the physicians of the hospitals, in order that this substance may be tested in actual epidemic cholera. The preparation of the salt is very easy; it is sufficient to place uric acid in contact with canstic ammonia and evaporate the product.

It will be well to select for the first trials cases of a mild type, afterwards those of more severity. It may probably be necessary to administer it in larger doses than those which I have used in European cholera." J.

